# Soil microbiome transplantation to enhance the drought response of *Salvia officinalis* L.

**DOI:** 10.3389/fmicb.2025.1553922

**Published:** 2025-03-12

**Authors:** Renée Abou Jaoudé, Francesca Luziatelli, Anna Grazia Ficca, Maurizio Ruzzi

**Affiliations:** Department for Innovation in Biological, Agrofood and Forest Systems (DIBAF), University of Tuscia, Viterbo, Italy

**Keywords:** microbiome transplantation, plant growth-promoting rhizobacteria, *Salvia officinalis* L., plant ecophysiology, leaf metabolomics, drought

## Abstract

**Introduction:**

Soil microbiome transplantation is a promising technique for enhancing plant holobiont response to abiotic and biotic stresses. However, the rapid assessment of microbiome-plant functional integration in short-term experiments remains a challenge.

**Methods:**

This study investigates the potential of three evergreen sclerophyll species, *Pistacia lentiscus* (PL), *Rosmarinus officinalis* (RO), and *Juniperus phoenicea* (JP), to serve as a reservoir for microbial communities able to confer enhanced tolerance to drought in Salvia officinalis cultivated under water shortage, by analyzing biomass production, plant phenotype, plant ecophysiological responses, and leaf metabolome.

**Results:**

Our results showed that the inoculation with the three rhizomicrobiomes did not enhance total plant biomass, while it significantly influenced plant architecture, ecophysiology, and metabolic responses. The inoculation with the JP rhizomicrobiome led to a significant increase in root biomass, resulting in smaller leaves and a higher leaf number. These morphological changes suggest improved water acquisition and thermoregulation strategies. Furthermore, distinct stomatal conductance patterns were observed in plants inoculated with microbiomes from PJ and PL, indicating altered responses to drought stress. The metabolome analysis demonstrated that rhizomicrobiome transplantation significantly influenced the leaf metabolome of *S. officinalis*. All three rhizomicrobiomes promoted the accumulation of phenolic compounds, terpenoids, and alkaloids, known to play crucial roles in plant defense and stress response. Five molecules (genkwanin, beta-ionone, sumatrol, beta-peltatin-A-methyl ester, and cinnamoyl-beta-D-glucoside) were commonly accumulated in leaves of inoculated sage, independently of the microbiome. Furthermore, unique metabolic alterations were observed depending on the specific inoculated rhizomicrobiome, highlighting the specialized nature of plant-microbe interactions and the possible use of these specific molecules as biomarkers to monitor the recruitment of beneficial microorganisms.

**Discussion:**

This study provides compelling evidence that microbiome transplantation can induce phenotypic and metabolic changes in recipient plants, potentially enhancing their resilience to water scarcity. Our findings emphasize the importance of considering multiple factors, including biomass, physiology, and metabolomics, when evaluating the effectiveness of microbiome engineering for improving plant stress tolerance.

## 1 Introduction

The plant-associated microbiome, a complex consortium of microorganisms inhabiting the phyllosphere, rhizosphere, and endosphere, constitutes a vast reservoir of genetic diversity that plays a crucial role in plant health and fitness (Trivedi et al., 2020; Bhattacharjee et al., 2022). This intricate microbial network provides many benefits to the host plant, including enhanced nutrient acquisition and suppression of phytopathogens, ultimately contributing to increased plant productivity and survival (Trivedi et al., 2020; Bhattacharjee et al., 2022). The composition and function of the plant microbiome are dynamically shaped by a complex interplay of factors, including host genotype, soil properties, developmental stage, microbial competition, and environmental stressors (Pérez-Izquierdo et al., 2019; Trivedi et al., 2020; Mahmud et al., 2021; Berruto and Demirer, 2024). Consequently, elucidating the mechanisms by which these microbial communities, mainly bacteria and fungi, confer beneficial effects on plant growth and development has become a major focus of research (Abou Jaoudé et al., 2023). Plant growth-promoting rhizobacteria (PGPR) are known for their ability to enhance the nutritional status of plants through diverse mechanisms. These mechanisms include direct nutrient provision (Fürnkranz et al., 2008; Moreau et al., 2019), the conversion of recalcitrant nutrients into forms that are available to plants (Lorenzi et al., 2022; Raymond et al., 2021; Kumawat et al., 2021; de Andrade et al., 2023), and the enhancement of nutrient uptake efficiency. The latter is achieved through increased root length (Mantelin et al., 2006; Apine and Jadhav, 2011; Ferreira Rêgo et al., 2014; Marín et al., 2021) and enhanced lateral root development (Mantelin et al., 2006; Vanegas and Uribe-Vélez, 2014; Azizi et al., 2022). Consequently, integrating microbial biotechnology, particularly by harnessing beneficial plant-microbe interactions, presents a promising strategy for optimizing plant fitness and productivity and mitigating future food security challenges.

Exploiting the plant microbiome’s potential has driven the emergence of microbiome engineering, a field focused on utilizing microorganisms for enhancing plant growth (Berruto and Demirer, 2024). This approach encompasses various strategies, including the application of single or consortia of probiotic microbial strains possessing specific growth-promoting traits, the use of host plants to recruit beneficial microbiome members selectively, the modification of soil properties to stimulate the growth and activity of desirable microorganisms and microbiome transplantation (Song et al., 2021). Microbiome transplantation involves transferring a microbial community, along with its associated functional capabilities, from a donor to a recipient host. This method has been shown to be a more comprehensive and resilient approach to promoting plant growth compared to single-strain inoculations (Jousset and Lee, 2023). This technique has emerged as a promising strategy to overcome the limitations associated with single-strain or consortia inoculations, which often face challenges regarding survival and efficacy within complex environmental settings (Ray et al., 2020; Park et al., 2023). Manipulation of the plant microbiome via transplantation can be achieved through various methods, including a “wash procedure” involving the inoculation of concentrated microbial communities obtained from healthy donor plants via centrifugation (Toju et al., 2018) or through direct transfer of soil from the donor plant’s rhizosphere (Howard et al., 2017). This manipulation ideally occurs during the initial stages of plant development to maximize the influence of the introduced microbiome on the recipient plant’s microbial community. However, microbiome transplantation has often proven to be a trial-and-error process with a high failure rate (Choi et al., 2020). This lack of consistent success is likely attributed to unpredictable interactions and coalescence processes between the transplanted microbiome and the recipient plant’s existing microbial community (Rillig et al., 2016), as well as potential incompatibilities between the introduced microbiome and the host plant itself (Jousset and Lee, 2023). These challenges underscore the need for further research to understand the complex dynamics of microbiome transplantation and improve its efficacy.

While microbiome transplantation offers significant potential, its success depends on carefully selecting and screening donor microbiomes. Current evaluation methods primarily rely on analyzing plant responses, such as disease suppression or biomass increase, often coupled with assessing changes in soil quality and microbiome composition. For example, Wei et al. (2019), in a study aiming at exploring how initial soil microbiome composition influences disease response in tomatoes, found that the presence of rare specific taxa, such as pathogen-suppressing *Pseudomonas* and *Bacillus* and high abundance of genes encoding non-ribosomal peptide and polyketide synthases (antimicrobial compounds) predict plant survival to the plant pathogenic *Ralstonia solanacearum* bacterium. The next tomato generation planted in these soils was analyzed for disease incidence. A decrease in visible symptoms demonstrated that microbiome-mediated plant protection could be transferred via soil transplantation. Jiang et al. (2022) found that the rhizosphere microbiome of resistant varieties was enriched for distinct and specific bacterial taxa associated with disease suppression. The microbiome transplant efficacy was quantified using source tracking analysis, i.e., DNA-based techniques to identify the specific types of microorganisms present in the rhizosphere of recipient plants. While the presence of specific taxa can provide valuable insights into the potential benefits of a microbiome, relying solely on plant biomass assessment and microbial composition analysis may provide an incomplete understanding of the intricate interplay between the transplanted microbiome and the host plant. Therefore, it is essential to integrate compositional analysis with functional studies that thoroughly investigate the dynamic interactions between the microbiome and the host to elucidate the functional mechanisms underlying microbiome-mediated plant growth promotion. This approach should examine gene expression, metabolome profiling, and physiological responses in both the plant and the microbiome, particularly during the early stages of development, which can be challenging to assess in slow-growing species. Furthermore, while microbiome composition is crucial for evaluating its safety, a comprehensive understanding of its functional integration with the host plant is paramount for harnessing its full potential to enhance plant growth, development, and stress resilience.

Drought is a primary cause of crop yield reduction, posing a significant threat to global food security (Reddy et al., 2004; Gupta et al., 2020). Plants have evolved various mechanisms to cope with water scarcity, including (1) modifications in root architecture to enhance water uptake, (2) stomatal closure regulated by hormonal signals to minimize water loss, (3) the accumulation of metabolites to adjust osmotic pressure, (4) the dissipation of excess energy and the synthesis of metabolites to mitigate oxidative stress arising from the accumulation of reactive oxygen species (ROS) due to an impaired consumption between energy production (NADPH-H^+^ and ATP) in the light reaction and consumption in the Calvin cycle (Selmar and Kleinwächter, 2013; Caser et al., 2019; Kollist et al., 2019; Gupta et al., 2020). Consequently, drought causes a reprogramming of plant metabolism, affecting enzyme activity, substrate availability, and the demand for specific stress-responsive primary (sugars, polyols, amino acids) and secondary metabolites (Kumar et al., 2021).

*Salvia officinalis* L. (sage) is a valuable medicinal and aromatic shrub native to the Mediterranean region, an environment characterized by prolonged periods of water deficit and high temperatures (Savi et al., 2016; Valkovszki et al., 2023). This species is renowned for its essential oil, rich in terpenoids and phenolics, contributing to its numerous biological activities (Grdiša et al., 2015). While generally tolerant to water scarcity, severe drought can negatively impact *Salvia* species (Caser et al., 2019; Khodadadi et al., 2023; Li et al., 2023).

This study explores the potential of leveraging specialized rhizosphere microbiomes from stress-tolerant Mediterranean aromatic plants (*Rosmarinus officinalis* L., *Pistacia lentiscus* L., and *Juniperus phoenicea* L.) to enhance the resilience and productivity of *Salvia officinalis* L. (sage) under water-limited conditions. Plants thriving in marginal environments often harbor unique microbial communities with enhanced adaptive capabilities shaped by co-evolutionary processes under challenging conditions (Meena et al., 2017). These specialized microbiomes represent an untapped resource for discovering novel microbial biostimulants with the potential to overcome the limitations of existing products and contribute to sustainable agricultural practices. Furthermore, the rhizomicrobiome of medicinal plants in such environments exhibits rich microbial diversity, driven by the selective pressure exerted by root exudates and secondary metabolites (Jabborova et al., 2024), increasing the likelihood of harboring rare taxa with beneficial traits.

This study employs a multidisciplinary approach to (1) evaluate the ability of the transplanted microbial communities to enhance drought resistance in sage and (2) elucidate the functional integration of these microbiomes with the host plant by investigating the architectural, ecophysiological, and metabolic responses of *S. officinalis* under severe drought conditions. The goal is to develop effective tools for early identification of beneficial microbiomes or specific strains, enabling their selection before the full microbiome composition is defined, particularly in slow growing species subjected to stress.

## 2 Materials and methods

### 2.1 Soil sampling and microbiome extraction

Soil samples (0–15 cm depth) were collected under the canopy of typical Mediterranean maquis plants in the Nature Park Porto Conte (Sardinia, Italy) during late summer 2023, following a prolonged period of drought. Three soil samples were, respectively, extracted with a soil corer under the canopy of *Rosmarinus officinalis* L. (RO), *Juniperus phoenicea* L. (JP), and *Pistacia lentiscus* L. (PL). The soils were refrigerated during the transportation, sieved at 2 mm in the laboratory, pooled to obtain one sample per species, and finally stored at −20°C until further use. Fifty grams of stored soils were hydrated with 12–15 mL of 1:10 diluted Luria Bertani broth (LB, 10 g l^−1^ tryptone, 5 g l^−1^ yeast extract, 10 g l^−1^ NaCl) and incubated at 25°C for approximately 72 h. After reactivation of the microbial cell metabolism, the soil samples were added with 125 mL 0.1% peptone water (10 g l^−1^ peptone, 5 g l^−1^ NaCl) and homogenized using a BagMixer 400S blender (Interscience, Puycapel, France) set at speed 1, for 2 min. The soil extract was first centrifuged at 1,000 rpm for 10 min to remove soil sediments. The suspension was extracted and then centrifuged at 10,000 rpm for 10 min. The supernatant was discharged, and the cellular pellet was resuspended in 1 mL peptone water. To multiply the mixed populations composing the microbiomes, aliquots of 250 μL of the high-density cell suspension were transferred on sterile membranes (0.22 μm) posed at the center of sterile plates containing M9 agar medium (Miller, 1972) amended with yeast extract (0.05% w/v). The dishes were incubated for 18 h at 28°C. The cells were recovered from filters by washing with Peptone Water and then diluted in sterile water to reach an optical density OD = 1.

### 2.2 Plant experimental setup

Seeds of *S. officinalis* L. were surface sterilized by 10-min immersion in sodium hypochlorite (NaClO) solution (50% v/v) amended with Twin 20 (0.025% v/v). Seeds were rinsed 10 times with 2 mL of sterile water after sanitization. Sterilized seeds were sown on a sterile substrate composed of two parts peat and one part vermiculite in a germination chamber maintained at 26°C with a photon flux density of 200 μmol photons m^−2^s^−1^ for 11 days and a photoperiod of 14/10 h (light/dark). Twenty seedlings of uniform size were then selected and divided into four groups. Each group of five seedlings was transplanted into 55-liter growth pots containing 40 L of a sterile peat, perlite, and vermiculite substrate (2:1:1 v/v/v) placed in a grow tent (Mars Hydro EU, Ginsheim-Gustavsburg, Germany) equipped with a lamp Mars Hydro Smart FC 3000 Samsung LED Grow Light powered by Samsung LM301B led. The temperature was set to 26°C, and a ventilation system (DF150A, Inline Duct Fan, Mars Hydro EU) guaranteed an air exchange in the tent. The photosynthetic photon flux density (PPFD) was set to 350 μmol photon m^−2^ s^−1^ while the photoperiod was kept unchanged. The plantlets were left to adapt to the new conditions for 24 h, then PPFD was increased to 450 μmol photon m^−2^ s^−1^ and kept constant until the end of the experiment. After the adaptation period, each pot was irrigated with 4 L of sterilized deionized water, added with 0.1% (v/v) 0.2 μm filtered nutrient solution A (B’cuzz, Atami B.V., Rosmalen, The Netherlands) containing K_2_O 4.7%, CaO 3.8%, MgO 1.3%, SO_3_ 0.11%, Fe 0.04% and N 4.9% (calcium and ammonium nitrate salts); 0.1% (v/v) filtered nutrient solution B (B’cuzz, Atami B.V., Rosmalen, The Netherlands) containing P_2_O_5_ 4.1%, K_2_O 5.71%, B 0.01%, Mn 0.03%, Mo 0.001%, Zn 0.039%. The relative soil water content (SWC_R_) was 25%. All pots were irrigated once a week with 1 liter of nutrient solution to keep the SWC_R_ stable over time.

**Figure 1:**
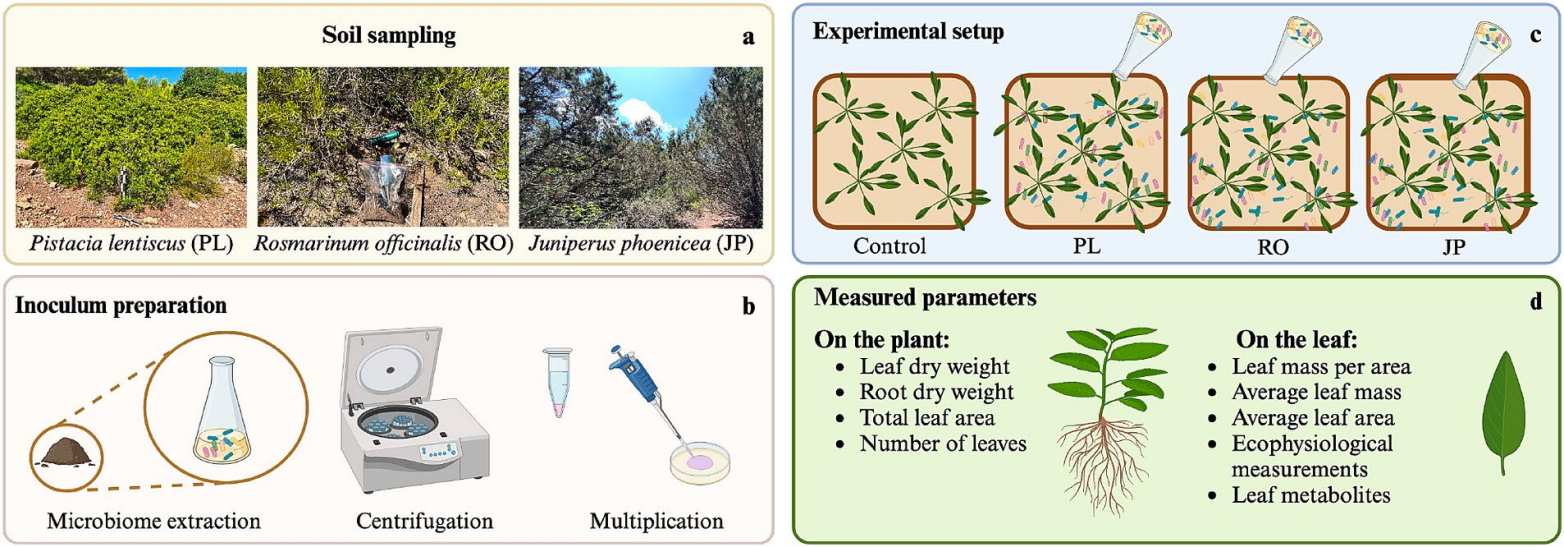
Rhizosphere microbiome transplantation procedure. **(a)** Soil collection from the rhizosphere of *Rosmarinus officinalis* (RO), *Pistacia lentiscus* (PL), and *Juniperus phoenicea* (JP). **(b)** Extraction, multiplication, and inoculum preparation of microbiomes. **(c)** Experimental setup of *Salvia officinalis* inoculation. **(d)** List of parameters that were measured at the level of the plant and the leaf.

Pots were assigned to one of four treatments. At DAT1 (Day After Treatment), one pot (C) was not inoculated, while the others were inoculated with one of the three rhizomicrobiomes (RO, JP, and PL). In the inoculated treatments, 15 mL of the diluted (OD = 1) cell suspension was applied to the soil around the base of the plant stem, in the vicinity of the root system, while 15 mL of sterile water was provided to each plant in the C treatment. After 2 weeks, a second inoculum was prepared, and the same plants were inoculated. Treatments were performed on DAT14 to promote greater colonization of the roots (Figure 1).

### 2.3 Plant biomass and growth parameters

The experiment was terminated 30 days post-inoculation. This duration provided sufficient time for the development of new leaves exhibiting specific anatomical and structural adaptations and ensured leaf wilting and senescence, induced by the deliberately low soil water content, did not occur prior to collecting leaf material for metabolomic analysis. At DAT30, the plants were harvested and separated into leaf and root fractions. The fresh weight of each fraction was recorded (RFW: root fresh weight; LFW: leaf fresh weight). The total fresh weight (TFW) was calculated as the sum of RFW and LFW. The leaves were arranged on a flatbed scanner for digital image acquisition. ImageJ software (version 1.53 t, Wayne Rasband and contributors, National Institutes of Health, United States) was employed to analyze the images, determining total leaf area (TLA) and leaf number (LN) per plant. The average leaf surface area (ALA) was then calculated (ALA = TLA/LN). Subsequently, subsamples of plant roots and leaves were collected and subjected to oven drying using a Sartorius MA 100 moisture analyzer (Göttingen, Germany). The total root dry weight (RDW) was determined by weighting roots after desiccation. The total leaf dry weight (LDW) was estimated by establishing the relationship between fresh and dry weight for a subsample of leaves from each plant belonging to the four treatments (LDW_C_ = LFW_C_ × 0.1701 + 0.04, *R*^2^ = 0.9998, *F* = 4847.15, *p* < 0.001; LDW_RO_ = LFW_RO_ × 0.1681 + 0.0448, *R*^2^ = 0.931, *F* = 135.01, *p* < 0.01; LDW_JP_ = LFW_JP_ × 0.0915 + 0.1295, *R*^2^ = 0.9961, *F* = 253.76, *p* < 0.05; LDW_PL_ = LFW_PL_ × 0.1725–0.0489, *R*^2^ = 0.9978, *F* = 447.15, *p* < 0.05). The total plant dry weight (TDW) was determined by the sum of root dry weight (RDW) and leaf dry weight (LDW). The average leaf biomass (ALB) was subsequently calculated as (ALB = LDW/LN). Finally, leaf mass per area (LMA) was determined as the ratio of total leaf area to leaf dry weight.

### 2.4 Ecophysiological measurements

Stomatal conductance (g_s_) and electron transport rate (ETR) were taken at DAT7, DAT14, DAT21, and DAT 28 on the first fully expanded leaf from the apical bud of each plant for each treatment, using a LI-600 porometer (Li-Cor, Lincoln, Oregon, United States) with a flow rate of 150 μmol s^−1^.

### 2.5 Leaf metabolite profiling

Leaf tissue samples designated for elemental analysis were pooled, homogenized, and subjected to cryogenic preservation using liquid nitrogen and stored at −20°C. Metabolite extraction was performed using an acidified 80% methanol solution, following the protocol established by Paul et al. (2019). The samples were extracted by an Ultra-Turrax (Ika T-25; Staufen, Germany) homogenizer. The samples were centrifuged, and the supernatant was filtered using a 0.22 μm cellulose membrane. Filtered extracts were subjected to an untargeted metabolomic analysis conducted by oloBion Laboratory (Barcelona, Spain). Metabolite identification and quantification were performed according to the methodology described by Bonini et al. (2020).

### 2.6 Statistical analysis

To test the effect of each microbiome inoculation on plant biomass, phenotype, and physiological parameters, mean values for each treatment were subjected to a two-sample *t*-test on the website www.socscistatistics.com. Statistically significant differences at a significance level of *p* ≤ 0.05 were considered.

## 3 Results

### 3.1 Effect of microbiome inoculation on leaf metabolome

A comprehensive untargeted metabolomic analysis of the leaf metabolome of *Salvia officinalis* cultivated under water deficit was conducted to evaluate the effect of three microbial consortia extracted from the rhizosphere of *Rosmarinus officinalis*, *Juniperus phoenicea*, and *Pistacia lentiscus*. This analysis revealed 359 metabolites whose abundance varied among inoculated and non-inoculated plants. Two hundred ninety-eight metabolites were distributed across 39 superclasses and 99 distinct Natural Products (NP) Classification classes. The most prevalent classes included a significant presence of diterpenoids (*n* = 27), with a notable abundance of abietane diterpenoids (*n* = 8) and gibberellins (*n* = 7). Another prominent class was flavonoids (*n* = 22), comprising flavones (*n* = 10), flavonols (*n* = 7), and flavonones (*n* = 5). In addition, monoterpenoids (*n* = 24) and phenylpropanoids (*n* = 22) were identified in substantial quantities. Within the phenylpropanoid class, cinnamic acids and their derivatives accounted for 90% of the observed metabolites (Supplementary Figure S1).

**Figure 2:**
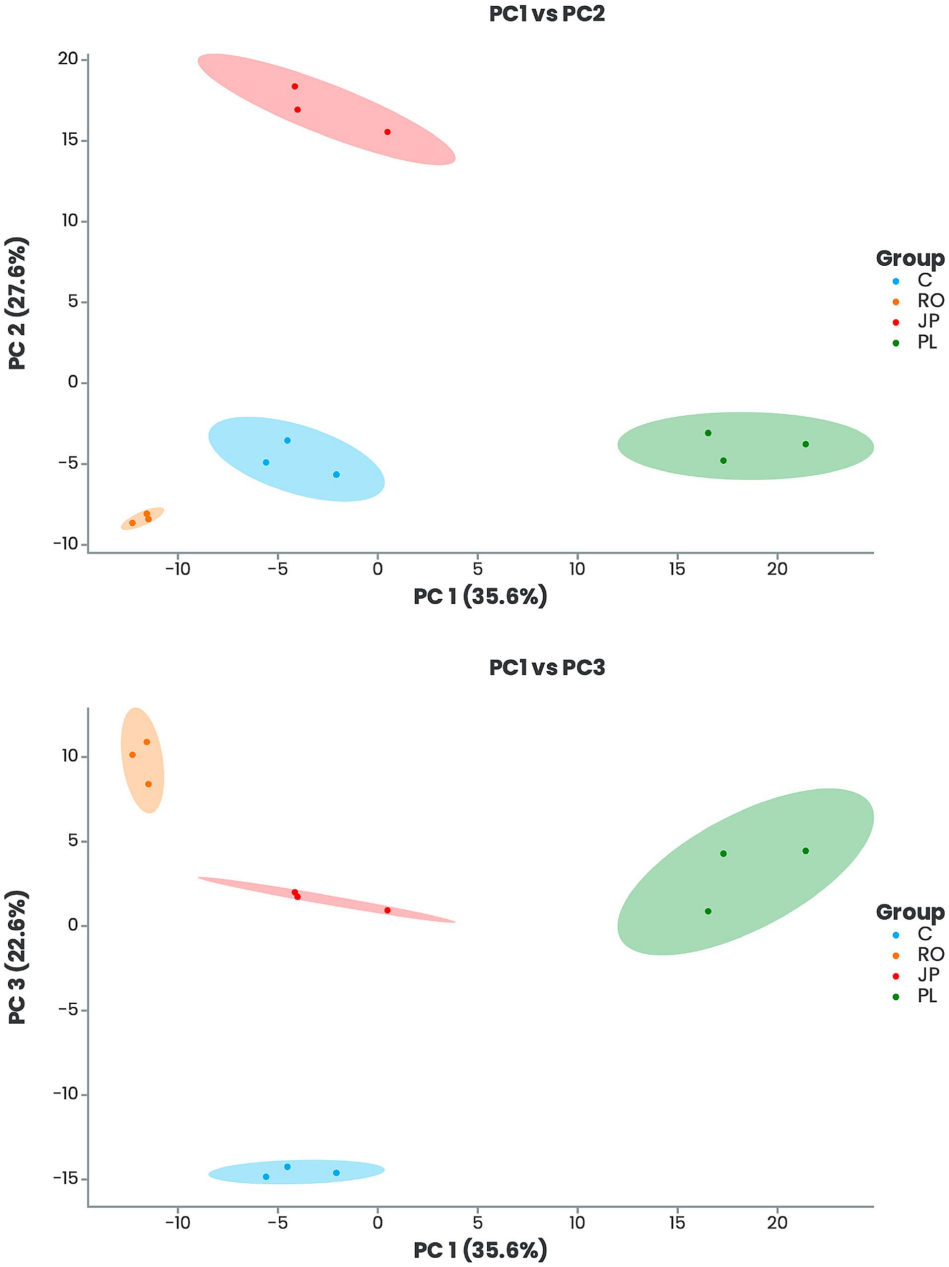
Principal component analysis (PCA) score plots of leaf metabolic profiles of inoculated and non-inoculated *S. officinalis* grown under low-water regime conditions. PC1-PC2 is shown in the upper panel, and PC1-PC3 in the lower panel. Plants were inoculated with the rhizosphere microbiomes from *R. officinalis* (RO), *P. lentiscus* (PL), and *J. phoenicea* (JP). (C) Non-inoculated plants.

A comparative analysis of leaf metabolome datasets was conducted using Principal Component Analysis (PCA). The study revealed that the first three principal components accounted for 85.8% of the total variance, with PC1, PC2, and PC3 contributing 35.6, 27.6, and 22.6%, respectively (Figure 2). The PCA demonstrated a clear separation between inoculated and non-inoculated plants and among the various treatments (Figure 2). The results indicated that plants inoculated with *P. lentiscus* were distinctly separated from the non-inoculated (C) and the other groups along PC1. In contrast, those inoculated with *J. phoenicea*- and *R. officinalis*-microbiome were separated along PC2 and PC3, respectively (Figure 2).

**Figure 3:**
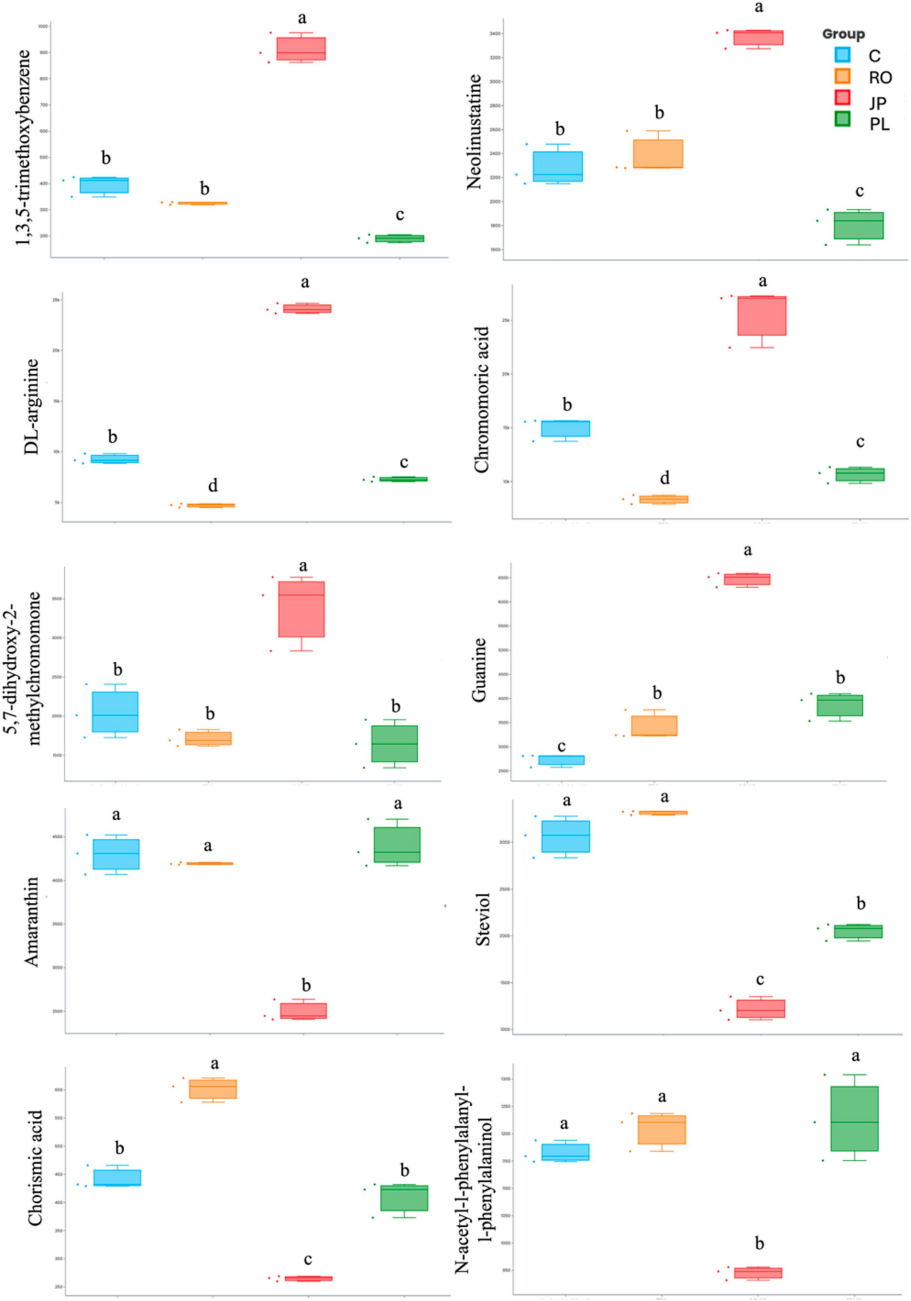
Box plot visualization of the abundance of the top 10 important features that explain the PCA differences in the leaf metabolome of microbiome-inoculated and non-inoculated (C) plants. Plants were inoculated with the rhizosphere microbiomes from *R. officinalis* (RO), *P. lentiscus* (PL), and *J. phoenicea* (JP). Distinct lowercase letters denote statistically significant variations (*p* < 0.05) among treatments for individual parameters, assessed independently for each parameter (*n* = 3).

The 10 most important features determining significant separation among treatments are reported in Figure 3. A total of six metabolites were found to be significantly more abundant in the leaf tissues of plants inoculated with the microbiome extracted from *J. phoenicea*, including 1,3,5-trimethoxybenzene, neolinustatine, DL-arginine, chromomoric acid, 5,7-dihydroxy-2-methylchomone, and guanine. In contrast, one metabolite (chorismic acid) was more abundant in the leaf tissue of *R. officinalis*-inoculated plants. Furthermore, eight metabolites, whose abundance exhibited a significant decrease in the leaf tissues of inoculated plants compared to non-inoculated plants, were identified. Two of these (DL-arginine and chromomoric acid) were detected in plants inoculated with the microbiome extracted from *R. officinalis*. Four (amaranthine, chorismic acid, N-acetyl-1-phenylalanyl-1-phenylalaninol, and steviol) were detected in plants inoculated with the microbiome extracted from *J. phoenicea*, and five (1,3,5-trimethoxybenzene, neolinustatine, DL-arginine, chromomoric acid, and steviol) were detected in those inoculated with the microbiome from *P. lentiscus* (Figure 3).

Chemical enrichment analysis of inoculated and non-inoculated datasets showed a complex variation in the leaf metabolite content due to inoculation with rhizosphere microbiomes (Supplementary Table S3). Data analysis revealed significant variations (*p* ≤ 0.05) in the relative abundance of about one-third of the metabolites (94 in plants inoculated with the microbiome extracted from *J. phoenicea* -, 96 in plants inoculated with the microbiome extracted from *P. lentiscus*, and 112 in plants inoculated with the microbiome extracted from *R. officinalis*) belonging to about 50 classes (Supplementary Table S1). The subset of metabolites whose abundance significantly increased ≥2-fold (*p* ≤ 0.05) in the leaf tissue of inoculated plants (compared to non-inoculated) varied between 15 (in plants inoculated with the microbiome extracted from *J. phoenicea*) and 26 (in plants inoculated with the microbiome extracted from *R. officinalis*) and was equal to 24 in plants inoculated with the microbiome extracted from *P. lentiscus* (Supplementary Table S2). Several were classified as flavonoids and phenylpropanoids (Supplementary Table S2). The flavonoid-related compounds corresponded to 2 (in plants inoculated with the microbiome extracted from *J. phoenicea*) to 7 (in plants inoculated with the microbiome extracted from *R. officinalis*) of the totals, with one compound (genkwanin) upregulated in all inoculated plants (fold change between 2.3- and 5.1-fold) and one (diosmetin) only in plants inoculated with the microbiomes extracted from *R. officinalis* and *P. lentiscus* (Supplementary Table S2). The number of phenylpropanoid-related compounds varied between 2 (in plants inoculated with the microbiome extracted from *J. phoenicea*) and 4 (in plants inoculated with the microbiome extracted from *P. lentiscus*), with one compound (cinnamoyl-beta-D-glucoside) upregulated in all inoculated plants (fold change between 2.1- and 2.9-fold) and one (coniferaldehyde glucoside) whose abundance significantly increased only in plants inoculated with the microbiome extracted from *J. phoenicea* and *R. officinalis*. Data analysis also revealed three additional metabolites whose abundance significantly increased ≥2-fold (*p* ≤ 0.05) in inoculated sages independently of the microbiome that was used (Supplementary Table S2). These metabolites belonged to 3 superclasses, including (1) apocarotenoids (beta-ionone; fold change between 2.3- and 2.7-fold), (2) lignans (beta-peltatin A methyl ether; fold change between 2.4- and 4.0-fold), and (3) isoflavonoids (sumatrol; fold change between 2.5- and 3.8-fold).

For 12 metabolites reported in Supplementary Table S2 (JP1-3, PL1-3, and RO1-6), the chemical enrichment analysis also revealed upregulation in the one-to-one comparison between datasets of differentially inoculated plants. The number of metabolites whose increase was associated specifically with one of the treatments ranged from 3 [in plants inoculated with the microbiome extracted from *J. phoenicea* (JP1-3) and *P. lentiscus* (PL1-3)] to 6 (in plants inoculated with the microbiome extracted from *R. officinalis* (RO1-6); Supplementary Table S2). The relative abundance of these metabolites changed up to 7.3-fold (5,7-dihydroxy-2-(4-hydroxy-3-methoxyphenyl)-4-oxo-4H-chromen-3-yl hexopyranoside (RO1), trans-2-hydroxycinnamic acid (RO3), and coumarin (RO4); Supplementary Table S3).

With two exceptions, RO1 [5,7-dihydroxy-2-(4-hydroxy-3-methoxyphenyl)-4-oxo-4H-chromen-3-yl hexopyranoside] and RO5 [6-methoxyluteolin], a more pronounced difference in their relative abundance was observed when comparing the leaf metabolome datasets of sages inoculated with the microbiomes extracted from *R. officinalis* and *P. lentiscus*. The range of the fold change values varied from 3.5- [2-(8-hydroxy-2-oxotridecyl)-6-oxopyran-4-olate (PL2 vs. RO)] to 7.3-fold [trans-2-hydroxycinnamic acid (RO3 vs. PL) and coumarin (RO4 vs. PL)] (Supplementary Table S3). For all 12 metabolites reported in Supplementary Table S3, the range of the fold change increase between inoculated and non-inoculated plants was comprised between 2.2 (JP3, sucrose-2-(2-methyl)butyryl-4-(2-methyl)butyryl-3-(4-methyl)hexanoyl-6-isobutyrate) and 3.4 (RO4, coumarin).

Sage inoculation with rhizosphere microbiomes also resulted in a significant reduction (fold change ≤ 0.5-fold; *p* ≤ 0.05) in the relative abundance of 9 (in plants inoculated with the microbiome extracted from *J. phoenicea* and *R. officinalis*) and 10 (in plants inoculated with the microbiome extracted from *P. lentiscus*) metabolites compared to the non-inoculated plants (Supplementary Table S4). No compound was identified as being shared among the three datasets. The most significantly downregulated superclass of molecules was diterpenoids, comprising approximately one-third of the downregulated compounds in the leaf metabolome of plants inoculated with the microbiome extracted from *J. phoenicea* and *P. lentiscus* (Supplementary Table S4). 3beta,15,16-trihydroxydolabrene, one of the three metabolites downregulated in plants inoculated with the microbiome extracted from *J. phoenicea* and *P. lentiscus*, belongs to this superclass. In contrast, metabolites that exhibited a significant decrease in plants inoculated with the microbiome extracted from *J. phoenicea* and *R. officinalis* were classified as steroids (19-hydroxytestosterone) and sphingolipids (sphingosine 1-phosphate; Supplementary Table S3). Notably, no compound was identified as being shared between the downregulated microbiomes from *P. lentiscus* and *R. officinalis* datasets.

### 3.2 Effect of microbiome inoculation on biomass production and plant structure

**Figure 4:**
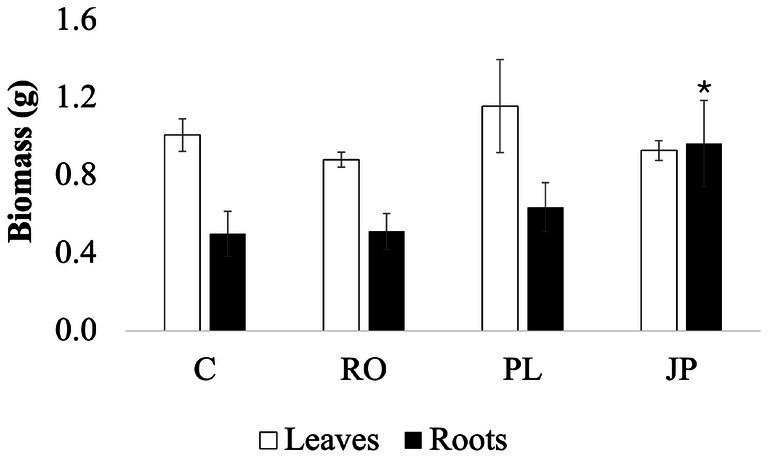
Effect of the microbiome transplantation on *S. officinalis* growth under low-water regime conditions. Aboveground biomass (white bars) and belowground biomass (black bars) were determined at DAT30. Data are presented as means ± standard errors. Asterisks denote statistically significant variations (*p* < 0.05) among treatments for individual parameters, assessed independently for each parameter (*n* = 5). (C) Non-inoculated sages. (JP), (PL), and (RO), plants inoculated with the rhizosphere microbiomes from *J. phoenicea* L., *P. lentiscus* L., and *R. officinalis* L., respectively.

Sage inoculation with the three microbiomes did not alter total plant biomass. On DAT30, the average total plant biomass was recorded at 1.70 ± 0.3 g, with no significant differences in aboveground biomass (1.04 ± 0.2 g) among the inoculated sage plants (Figure 4). However, significantly higher (*p* < 0.05) root biomass was found in plants inoculated with the microbiome extracted from *J. phoenicea* (0.97 ± 0.2 g) compared to the non-inoculated sages (0.50 ± 0.1 g). In contrast, root biomass in plants inoculated with the microbiome extracted from *R. officinalis* (0.51 ± 0.1 g) and *P. lentiscus* (0.64 ± 0.1 g) inoculated plants was not significantly affected by the treatment (Figure 4).

Sage inoculation with the microbiome extracted from *J. phoenicea* also altered the plant phenotype. Although the total leaf area was not affected by the inoculation treatments, remaining similar in both inoculated and non-inoculated plants (on average, 135.4 ± 27.2 cm^2^; Table 1), a significant increase in the number of leaves was observed in sage inoculated with the microbiome extracted from *J. phoenicea* (47.5 ± 7.3 leaves per plant) compared to non-inoculated sages (21.5 ± 2.4 leaves per plant, *p* < 0.05). In contrast, no modification in this parameter was observed in sage inoculated with the microbiome extracted from *R. officinalis* and *P. lentiscus* (26.3 ± 5.0 and 24.3 ± 6.7 leaves per plant, respectively; Table 1).

**Table 1:** Responses of *S. officinalis* leaf traits to microbiome transplantation.

	TLA (cm^2^)		LN		ALA (cm^2^)		ALM (g)		LMA (g m^−2^)	
Treatment	Mean	s.e.	Mean	s.e.	Mean	s.e.	Mean	s.e.	Mean	s.e.
C	123.9	23.2	21.5	2.4	5.6	0.5	0.018	0.002	31.9	3.0
RO	111.4	20.7	26.3	5.0	4.3	0.1	0.015	0.002	35.7	4.8
PL	125.5	29.5	24.3	6.7	5.4	1.1	0.027	0.009	45.1	8.1
JP	180.7	35.5	47.5	7.3*	3.8	0.4*	0.009	0.001**	23.5	2.6*

Total leaf area (TLA), number of leaves (LN), average leaf area (ALA), average leaf mass (ALM), and leaf mass per area (LMA) were measured at DAT30. The data are expressed as the mean ± standard error (s.e.). Asterisks denote statistically significant variations for individual parameters (*n* = 5; *p* < 0.05) between the non-inoculated sages (C) and plants inoculated with the rhizosphere microbiome from *R. officinalis* L. (RO), *P. lentiscus* L. (PL), and *J. phoenicea* L. (JP).

The leaf area exhibited a significant response only in sages inoculated with the microbiome extracted from *J. phoenicea*. Specifically, the leaf area was significantly (*p* < 0.05) lower in sages inoculated with the microbiome extracted from *J. phoenicea* (3.8 ± 0.4 cm^2^) compared to non-inoculated sages (5.6 ± 0.5 cm^2^). In contrast, inoculation with the microbiomes extracted from *P. officinalis* and *P. lentiscus* resulted in average leaf area values similar to those of non-inoculated sages, averaging 4.8 ± 0.6 cm^2^ (Table 1). Furthermore, an examination of average leaf mass revealed a statistically significant (*p* < 0.01) decrease in plants inoculated with the microbiome extracted from *J. phoenicea* (0.009 ± 0.001 g) compared to non-inoculated sages (0.018 ± 0.002 g, Table 1). However, no substantial differences were observed in the average leaf mass of plants inoculated with the microbiome extracted from *R. officinalis* (0.015 ± 0.002 g) or *P. lentiscus* (0.027 ± 0.009 g) inoculated plants (Table 1). The leaf mass per area exhibited a significant response only in sages inoculated with the microbiome extracted from *J. phoenicea*. Specifically, the ratio of leaf mass to leaf area was significantly lower (*p* < 0.05) in sages inoculated with the microbiome extracted from *J. phoenicea* (23.6 ± 2.6 g m^−2^) compared to non-inoculated sages (31.9 ± 3.0 g m^−2^; Table 1). Inoculation with the microbiomes extracted from *R. officinalis* and *P. lentiscus* did not affect the ratio, with leaf mass per area measuring 35.7 ± 4.8 and 45.1 ± 8.1 g m^−2^, respectively, similar to the levels observed in non-inoculated plants (Table 1).

### 3.3 Effect of microbiome inoculation on leaf ecophysiology

**Figure 5:**
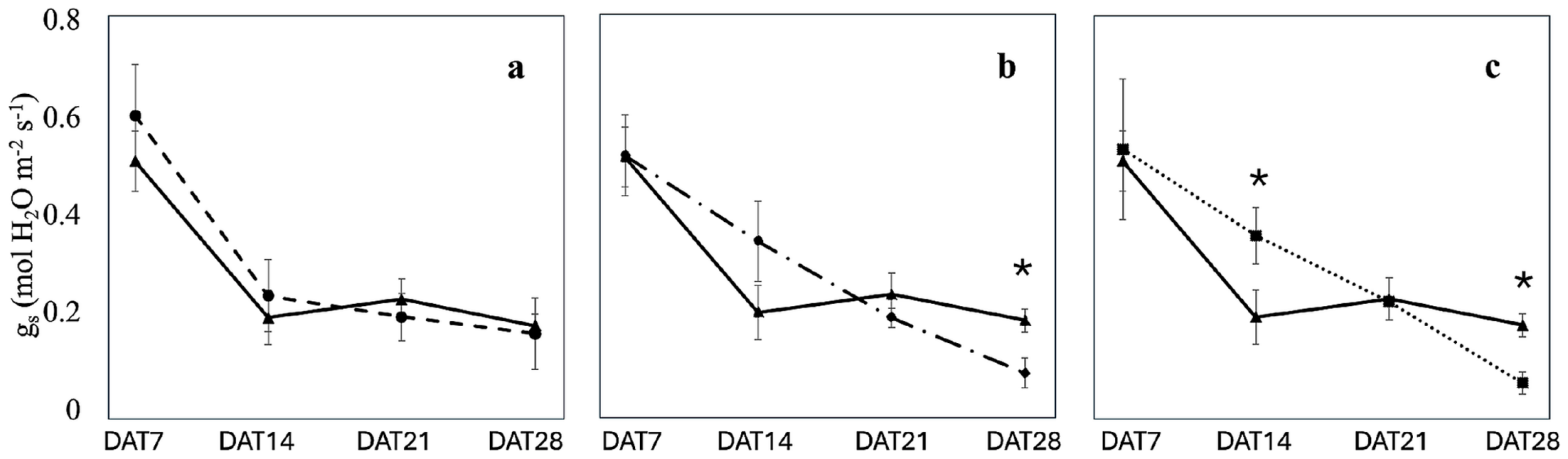
Effect of the microbiome transplantation on the stomatal conductance (*g_s_*) of *S. officinalis* grown under low-water regime conditions. The measurements were conducted weekly for 4 weeks (DAT7, DAT14, DAT21, and DAT28) on non-inoculated (C) and plants inoculated with the rhizosphere microbiomes from *R. officinalis* L. (RO; **a**), *P. lentiscus* L. (PL; **b**), and *J. phoenicea* L. (JP; **c**). Data are presented as means ± standard errors. Asterisks denote statistically significant variations (*p* < 0.05) between C and inoculated plants, assessed independently for each microbiome (*n* = 5).

Leaf stomatal conductance and electron transport rate in plants inoculated with the three microbiomes were measured weekly over a four-week period. The results indicated that inoculation with the three microbiomes did not result in any significant changes in stomatal conductance on DAT7 (0.55 ± 0.10 mol H_2_O m^−2^ s^−1^) and DAT21 (0.21 ± 0.03 mol H_2_O m^−2^ s^−1^; Figure 5). However, on DAT 14, a significant (*p* < 0.05) increase in stomatal conductance was observed in plants inoculated with the microbiome extracted from *J. phoenicea* (0.37 ± 0.06 mol H_2_O m^−2^ s^−1^) compared to non-inoculated sages (0.20 ± 0.06 mol H_2_O m^−2^ s^−1^; Figure 5). On DAT 28, both plants inoculated with the microbiomes of *P. lentiscus* and *J. phoenicea* exhibited reduced stomatal conductance compared to non-inoculated plants (0.07 ± 0.03 mol H_2_O m^−2^ s^−1^ and 0.06 ± 0.02 mol H_2_O m^−2^ s^−1^, respectively; *p* < 0.05; Figure 5).

While stomatal conductance decreased over time, its dynamic varied among the treatments (Figure 5; Supplementary Table S5). In the absence of inoculation, a statistically significant (p < 0.05) decrease in stomatal conductance was observed on DAT14 compared to DAT7. The stomatal conductance remained constant through the subsequent two measurement dates (Supplementary Table S5). A similar trend was noted in sages inoculated with the microbiome of *R. officinalis* (Figure 5a; Supplementary Table S5). The decrease in stomatal conductance was less pronounced in plants inoculated with the *P. lentiscus* microbiome (Figure 5b; Supplementary Table S5). A significant (*p* < 0.05) decrease in stomatal conductance was observed on DAT21 compared to DAT7. However, stomatal closure significantly increased on DAT28, resulting in the lowest stomatal conductance value (Figure 5b; Supplementary Table S5). In contrast, in plants inoculated with the microbiome extracted from *J. phoenicea*, a significant decrease (*p* < 0.05) in stomatal conductance was observed only on DAT21 compared to DAT7 and DAT14 (Figure 5c; Supplementary Table S5).

**Figure 6:**
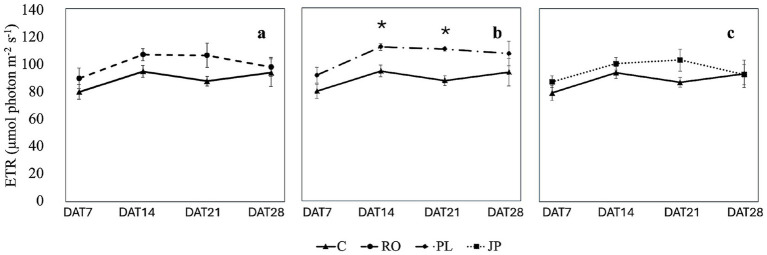
Effect of the microbiome transplantation on the Electron transport rate (ETR) of *S. officinalis* grown under low-water regime conditions. The measurements were conducted weekly for 4 weeks (DAT7, DAT14, DAT21, and DAT28) on non-inoculated (C) and plants inoculated with the rhizosphere microbiomes from *R. officinalis* L. (RO; **a**), *P. lentiscus* L. (PL; **b**), and *J. phoenicea* L. (JP; **c**). Data are presented as means ± standard errors. Asterisks denote statistically significant variations (*p* < 0.05) between C and inoculated plants, assessed independently for each microbiome (*n* = 5).

Inoculation did not modify the electron transport rate on DAT7 (93.1 ± 5.9 μmol photon m^−2^ s^−1^) and DAT 28 (104.3 ± 8.6 μmol photon m^−2^ s^−1^; Figure 6), regardless of the treatment. However, on DAT14 and DAT21, the electron transport rate was significantly higher (p < 0.05) in plants inoculated with the microbiome from *P. lentiscus* (119.0 ± 4.1 μmol photon m^−2^ s^−1^ and 117.3 ± 1.6 μmol photon m^−2^ s^−1^) compared to non-inoculated sages (110.8 ± 4.4 μmol photon m^−2^ s^−1^ and 93.5 ± 3.6 μmol photon m^−2^ s^−1^; Figure 6).

Except for plants inoculated with the microbiome extracted from *R. officinalis*, which showed no variation in electron transport rate over time, this parameter increased from DAT 7 to DAT14 in all treatment groups. However, no significant change was observed in DAT21 compared to the two preceding dates (Supplementary Table S5). By DAT28, the electron transport rate in plants inoculated with the microbiome extracted from *J. phoenicea* decreased to initial values, while it remained unchanged in the other treatment groups compared to previous dates (Supplementary Table S5).

## 4 Discussion

Microbiome engineering, achieved through the transplantation of microbial communities from donor to recipient plants, is a promising biotechnology with the potential to enhance plant traits and survival under biotic or abiotic stress. Panke-Buisse et al. (2015) utilized *Arabidopsis thaliana* Col. in a multi-generational experimental system to select soil microbiomes that induced earlier or later flowering times in their hosts. They demonstrated that distinct microbiota profiles were assembled by flowering time treatment, and subsequent inoculation with these microbial communities induced flowering time modifications in both *A. thaliana* and *Brassica rapa*. Moreover, microbiome transplantation has shown considerable potential in mitigating plant diseases (Kwak et al., 2018; Wei et al., 2019; Bziuk et al., 2022; Jiang et al., 2022; Khatri et al., 2024), facilitating plant growth in contaminated soils (Yergeau et al., 2015), and enhancing plant resilience to abiotic stress factors (Zolla et al., 2013). Beyond the scope of agricultural applications, microbiome-based interventions are gaining traction in farming settings and natural ecosystems. These interventions offer a promising strategy for restoring biodiversity and enhancing the resilience of wildlife and ecosystems. Notably, microbiome transplantation has been shown to improve tree growth and survival under drought and heat stress when recipient trees are inoculated with microbial communities from harsh environments (Allsup et al., 2023). This approach holds significant potential for mitigating climate change impacts, including projected reductions in winter rainfall of 15% by 2030 and 30% by 2070 (IPCC, 2022).

In this study, we evaluated the potential of three evergreen sclerophyll species, *P. lentiscus*, *R. officinalis,* and *J. phoenicea*, seasonally subjected to drought, to serve as a reservoir for microbial communities able to confer enhanced drought resistance traits to *S. officinalis* cultivated under water shortage, by analyzing biomass production, plant phenotype, and leaf metabolome. *S. officinalis* is a typical species inhabiting the Mediterranean Basin characterized by semi-arid soils, long-term decrease in water availability, and extremely high air temperatures and irradiance (Armada et al., 2013; Savi et al., 2016). Despite its tolerance to drought, *S. officinalis* is adversely affected by prolonged reductions in soil water potential. Grisafi et al. (2017) observed decreased stomatal conductance, net photosynthesis, and leaf area in *S. officinalis* under drought conditions. Savi et al. (2016) further demonstrated that the leaves of *Salvia* spp. exhibited a decline in water transport efficiency at water potential values that are more typical of mesophyte species than of xerophyte species. These observations highlight the complexity of drought responses in sage and suggest that introducing beneficial rhizomicrobiomes may offer a strategy to enhance its resilience to water scarcity, potentially mitigating the negative impacts of drought on physiological processes and growth. *S. officinalis* is a valuable species for revegetation programs in semiarid Mediterranean ecosystems. Enhancing plant establishment by directly applying bacterial inocula may benefit these efforts (Armada et al., 2013).

The composition of a microbiome is significantly influenced by the host plant (Santoyo, 2022), plant–plant interactions (Abou Jaoudé et al., 2024; Newberger et al., 2023), and environmental growth conditions (Postiglione et al., 2022). Idbella et al. (2022) identified differences in the composition of the rhizomicrobiomes of several Mediterranean plant species, including *P. lentiscus*, *J. phoenicea*, *Myrtus communis* L., *R. officinalis*, *Olea europaea* L., and *Euphorbia dendroides* L. They reported that soils associated with *P. lentiscus* L. exhibited the lowest nitrogen content and the highest abundance of free-living nitrogen-fixing bacteria. In a previous study on the phyllosphere microbiome of *P. lentiscus* L. collected from the same location utilized for rhizomicrobiome sampling in this study, Abou Jaoudé et al. (2024) highlighted the presence of numerous strains exhibiting a high tolerance to osmotic stress. These findings support the hypothesis that these microbiomes can thrive under similar environmental conditions and may be utilized in microbiome transplantation experiments.

The application of the three rhizomicrobiomes showed dissimilarities in sage biomass production and allocation, leaf number and morphology, leaf ecophysiological responses, and leaf metabolome compared to non-inoculated plants. While inoculation did not significantly alter total plant biomass regardless of the treatment, sage inoculated with the microbiome extracted from *J. phoenicea* exhibited a notable increase in root biomass compared to non-inoculated controls (Figure 4). As Chieb and Gachomo (2023) reported, an increase in root surface in drought-stressed plants can enhance water and nutrient uptake and boost hydraulic conductivity, improving adaptation to water deficit conditions. The observed higher root biomass in sages inoculated with the microbiome extracted from *J. phoenicea* might be attributed to changes in hormonal signaling. PGPR can interfere with phytohormone signals and control root development (Ranjan et al., 2024). Mainly, auxin is involved in the emission of lateral root and root hairs, promoting nutrient uptake by increasing the root surface (Rivas et al., 2022). Many root-associated microbial strains have been shown to produce auxin (Keswani et al., 2020). Several researchers have demonstrated that the synthesis of this compound is essential for the plant–PGPR interaction, influencing both the phenotypic and transcriptional responses of the host plant (Spaepen et al., 2007; Luziatelli et al., 2020; Xu et al., 2023).

Inoculation with the rhizomicrobiome from *J. phoenicea* resulted in a reduction in the average leaf area, accompanied by an increase in leaf number to maintain a similar total leaf surface area (see Table 1). Individual cells’ size variation mostly depends on vacuole expansion through water uptake (Forouzesh et al., 2012). Consequently, reduced leaf size is generally associated with environments with limited water availability (Basal et al., 2005), as drought stress negatively affects leaf expansion (Gray and Brady, 2016). Smaller leaves possess a thinner boundary layer, promoting convective heat dissipation compared to bigger leaves (Leigh et al., 2017) and inducing faster water losses (Wang et al., 2019), positively influencing plant thermoregulation. Furthermore, similar to the behavior observed in compound leaves, the shedding of smaller leaves may help mitigate the effects of localized water stress. This process can prevent widespread hydraulic failure and minimize biomass loss. The reduction in leaf size observed in plants inoculated with the rhizomicrobiome from *J. phoenicea* compared to non-inoculated plants indicates an enhanced capacity for water availability, probably triggered by the increase in root biomass. As smaller leaves represent an advantage in arid environments where water conservation is crucial, we can hypothesize a better response of plants inoculated with the rhizomicrobiome from *J. phoenicea* to drought in the long term. Abate et al. (2021) demonstrated the importance of root hydraulics in drought resistance for *Salvia* species. They suggest that increased biomass allocation to the root system enhances the accumulation of reserves crucial for post-drought recovery. In our study, the observed modifications in leaf structure and root biomass in plants inoculated with the rhizomicrobiome from *J. phoenicea* could contribute to a more resilient response to water deficit, facilitating superior recovery and survival in plants inoculated with the rhizomicrobiome from *J. phoenicea* compared to non-inoculated plants under prolonged drought conditions. These results may explain the reduced leaf mass per area (LMA) observed in plants inoculated with the rhizomicrobiome from *J. phoenicea* (Table 1), a response contrary to that typically observed in plants under water deficit conditions (de Dato et al., 2013). High leaf mass per area represents a potential adaptation to stressful environments such as those characterized by a Mediterranean climate and is associated with increased leaf thickness and density, reducing mesophyll conductance (Niinemets, 1999; Flexas et al., 2008). The decrease in leaf thickness induced by the inoculation with the rhizomicrobiome from *J. phoenicea* may have shortened the mesophyll pathway for CO_2_ to carboxylation sites, thereby increasing mesophyll conductance and mitigating stomatal limitations to photosynthesis. Indeed, stomatal closure is a common and rapid plant defense to preserve water (Gupta et al., 2020): when turgor pressure changes in guard cells, stomatal closure is stimulated (Osakabe et al., 2014).

The leaf ecophysiological measurements demonstrated that microbiomes induced a different response to drought in inoculated plants. In sage plants not subjected to inoculation, stomatal conductance showed high values in DAT7 and decreased to about 20% (100 mmol H_2_O m^−2^ s^−1^) in DAT14, maintaining a constant value in the following 2 weeks (Figure 5). Plants inoculated with the microbiome extracted from *R. officinalis* exhibited a similar trend (Figure 5a). These observations are consistent with the findings of Raimondo et al. (2015), who reported comparable stomatal conductance trends and values in *Salvia* grown under similar experimental conditions. Similarly, Savi et al. (2016) observed a significant decline in stomatal conductance of *S. officinalis* growing in natural ecosystems from June to July and August, followed by an increase in September concurrent with elevated soil water potential. Reductions in stomatal conductance were also reported by Abate et al. (2021) in *S. officinalis* subjected to different water stress levels and subsequent recovery. Caser et al. (2019) showed that stomatal conductance reduction in *Salvia dolomitica* subjected to severe drought was associated with increased abscisic acid concentration compared to well-watered plants. In response to drought-induced stress, plants synthesize abscisic acid endogenously. This hormone acts as a signaling molecule and triggers the accumulation of ROS in the cytoplasm of guard cells and of Ca^2+^ in the cytosol, reducing turgor and inducing stomatal closure (Osakabe et al., 2014; Liu et al., 2022). In our study, the abundance of abscisic acid did not follow the same pattern of stomatal conductance, being higher in non-inoculated and in plants inoculated with the microbiome extracted from *J. phoenicea* compared to those inoculated with the microbiome from *R. officinalis* (data not shown) at DAT28, suggesting that *Salvia* spp. can differently respond to reduced leaf water potential induced by water deficit. The observed stomatal conductance response of *S. officinalis* is characteristic of anisohydric species, which prioritize maximizing stomatal conductance under high water availability and exhibit less stringent stomatal control than isohydric species (Raimondo et al., 2015). This behavior is attributed to a more moderate induction of abscisic acid biosynthesis under drought stress at the root level, resulting in the maintenance, rather than an increase, of abscisic acid concentration relative to leaf tissue water content (Gallé et al., 2013).

Unlike the non-inoculated plants and the plants inoculated with the rhizomicrobiome from *R. officinalis*, which showed a drastic decrease in stomatal opening at DAT14, plants inoculated with the rhizomicrobiome from *J. phoenicea* and *P. lentiscus* exhibited a continuous negative trend, culminating in significantly lower minimum stomatal conductance at DAT28 (Figures 5b, 6c). Notably, inoculation with the rhizomicrobiome from *J. phoenicea* mitigated the decline in stomatal conductance observed in non-inoculated and in plants inoculated with the microbiome extracted from *R. officinalis* at DAT14 (Figure 5b), potentially due to increased water availability resulting from greater root biomass. However, this mechanism does not explain the stomatal response observed in plants inoculated with the rhizomicrobiome from *P. lentiscus*, which instead exhibited a significant increase in electron transport rate at DAT14 and DAT21 compared to the control (Figure 6). This suggests that inoculation with the rhizomicrobiome from *P. lentiscus* may alleviate water stress through a different mechanism, independent of root biomass enhancement. Liu et al. (2019) reported similar findings, observing that *Sambucus williamsii* inoculated with *Acinetobacter calcoaceticus* X128 exhibited less pronounced reductions in stomatal conductance and assimilation rates compared to non-inoculated plants under drought stress. This observation suggests that the interaction between *A. calcoaceticus* X128 and *S. williamsii* triggers a drought-mitigating response. Akhtar et al. (2021) reported increased photosynthetic activity and stomatal conductance in drought-stressed *Triticum aestivum* inoculated with *Bacillus* sp. and *Azospirillum* strains, attributing these effects to enhanced activity of antioxidant enzymes, specifically peroxidase and catalase. Despite the higher CO_2_ assimilation rate, no increase in biomass was observed, potentially due to the energy demands associated with the production of secondary metabolites.

The analysis of the plant metabolic responses induced by inoculation can give important insights into the mechanisms of increased plant resistance or growth promotion under stress conditions. Data presented in Supplementary Figures S2, S3 and Supplementary Tables S1–S3 demonstrate that rhizomicrobiome transplantation significantly altered the leaf metabolome of sage subjected to water limitation. All three rhizomicrobiomes promoted the accumulation of molecules belonging to phenolic compounds, terpenoids and alkaloids, which can be valuable in regulating the plant response to water-limited conditions (Kumar et al., 2023). Phenolic compounds, specifically phenylpropanoids and flavonoids, deriving from the phenylpropanoid pathway (Deng and Lu, 2017), are plant secondary metabolites that contribute to scavenge ROS produced under drought stress and are, therefore, correlated to plant drought tolerance (Moradi et al., 2017). An increase in phenols with increasing drought stress was reported in *Salvia sinaloensis* subjected to moderate and severe drought (Caser et al., 2018). Higher polyphenol contents were also observed in *S. officinalis* under mild and severe water deficits (Bettaieb et al., 2011). Under stress conditions, plants often trade between growth and secondary metabolite production. The accumulation of these metabolites typically coincides with reduced biomass, reflecting a shift in carbon allocation. Resources are diverted toward the synthesis of protective compounds, potentially at the expense of growth processes (de Abreu and Mazzafera, 2005). An increase in phenolic compounds is a reported response observed in both PGPR-inoculated plants (Mashabela et al., 2022) and plants infected with pathogens (Garcia et al., 2018). Flavonoids can contribute to various plant defense responses (Deng and Lu, 2017). Increased flavonoid levels were also observed in plants primed with PGPR and infected with pathogens, serving as signatory biomarkers for induced resistance against pathogens (Tugizimana et al., 2019; Carlson et al., 2019; Mhlongo et al., 2021). Moreover, flavonoids and phenolic acids are recognized as major secondary metabolites exuded by plant roots (Mandal et al., 2010; Cesco et al., 2012). Mashabela et al. (2022) proposed that increased levels of these compounds in leaves could prime plants for enhanced defense responses against pathogens and that the exudation of these secondary metabolites by roots serves as a chemotactic strategy to recruit beneficial microbes, thereby influencing rhizosphere microbiome composition and promoting plant-microbe interactions. Moreover, all three rhizomicrobiomes promoted the accumulation of lipids and terpenoids across several classes, which can be valuable in regulating the plant response to water-limited conditions. An accumulation of these secondary metabolites, precisely monoterpenes and sesquiterpenes has been observed in sage under drought stress (Nowak et al., 2010; Caser et al., 2019). The synthesis of highly reduced compounds, like isoprenoids, phenols or alkaloids is pushed during water stress, to counterbalance the massive oversupply of NADPH+H^+^. Thus, the biosynthesis of alkaloids and monoterpenes, through the consumption of NADPH, may contribute to the decrease in the reducing status of the electron transport chain present during stress conditions (Yahyazadeh et al., 2018).

Five compounds accumulated in the leaf metabolome of all the inoculated plants, independently of the type of rhizomicrobiome that was used. These metabolites belonged to five distinct classes, confirming that: (1) the rhizomicrobiome transplanting affects the leaf metabolome at multiple levels; (2) there are some metabolites, whose abundance is specifically altered, that can be used as biomarkers to monitor if the plant has recruited beneficial microorganisms. We found an increase in genkwanin abundance in all inoculated plants. Genkwanin has antibacterial (Cottiglia et al., 2001) and radical scavenging activity (Kraft et al., 2003). An increase in genkwanin was observed in *R. officinalis* plants grown in the dune sand during the summer, suggesting that the specific synthesis of flavonoids is enhanced in response to environmental stress (Boscaiu et al., 2019). Another up-regulated phenolic compound in inoculated plants is the phenylpropanoid cinnamoyl-beta-D-glucoside, a molecule that derives from a trans-cinnamic acid reacting with a beta-D-glucose (Deshaies et al., 2022). Deshaies et al. (2022) investigated chitosan’s impact on wheat’s early metabolomic response to *Fusarium graminearum* infection. Their analysis revealed a downregulation of cinnamoyl beta-D-glucoside during infection. As cinnamic acids are precursors to lignans, compounds known to reinforce plant cell walls and hinder fungal penetration, the authors suggest that this downregulation may impair lignification as a defense mechanism against *F. graminearum*. This result suggests that the increase in the presence of cinnamoyl-beta D-glucoside in our study indicates a potential priming effect of the microbiomes on *S. officinalis* lignification, which can serve as defense mechanisms against pathogens but can also enhance structural resilience under drought stress (Choi et al., 2023). Among the molecules up-regulated in all inoculated plants, the apocarotenoid beta-ionone has been reported to increase in abundance in plants subjected to salt stress (Mehdikhanlou et al., 2021). Apocarotenoids, products of carotenoid breakdown, are compounds that serve as hormones, volatile aromas, and intracellular secondary messengers (McQuinn and Waters, 2024). These molecules have been reported to be regulators and precursors of protective compounds in response to variations of environmental water, associated with drought tolerance (Vieira et al., 2024). Beta-ionone has been proposed as one of the signals, together with salicylic acid and jasmonate, initiating systemic acquired resistance (Huded et al., 2023). Beta-ionone application in *Arabidopsis* triggered extensive transcriptomic reprogramming, affecting numerous genes involved in stress responses, growth regulation, hormone metabolism, pathogen defense, and photosynthesis, enhancing resistance to *Botrytis cinerea* (Felemban et al., 2024). Interestingly, the authors reported that beta-ionone shares many features with another signaling molecule, beta-cyclocitric acid, which elicits plant drought tolerance (D’Alessandro et al., 2019). These results indicate that the upregulation of common metabolites induced by inoculation of the microbiomes can enhance *S. officinalis* resistance to biotic stress and drought tolerance.

In addition to common alteration of the above-mentioned classes and metabolites, inoculation with distinct rhizomicrobiomes also resulted in variations in microbiome-specific classes of compounds and abundance of unique metabolites, underscoring the specialized nature of plant-microbes interactions. Lei et al. (2019) demonstrated that besides the same selecting forces being responsible for the assembly of the core rhizosphere microbiome, the bacterial community composition associated with six plant species is specific to the plant hosts, and the more phylogenetically distant the plant hosts, the more distinct their associated bacterial communities are. These findings can have implications for microbiome selection to enhance the production of exclusive plant metabolites under water shortage, because the targeted application of drought has been proposed as a strategy to improve the quality of medicinal plants (Selmar and Kleinwächter, 2013). Manipulating the plant microbiome may offer a complementary approach to further enhance this effect. More importantly, these metabolites can be used as biomarkers for assessing the establishment of plant-microbiome interactions.

The PCA analysis of the leaf metabolome datasets provided further evidence that inoculation with the three microbiomes significantly altered the profiles of detectable leaf metabolites compared to non-inoculated plants (Figure 2). The analysis of the abundance of the top 10 important features that explain PCA differences (Figure 3) showed differences among the treatments. Among the most abundant metabolites, DL-arginine and chromomoric acid concentrations were higher in plants inoculated with the rhizomicrobiome from *J. phoenicea* compared to all the other treatments. Arginine is accumulated in drought-tolerant clones of eucalyptus trees and sesame genotypes subjected to drought stress (You et al., 2019; Noleto-Dias et al., 2023). Moreover, this amino acid was found to reduce the lipid peroxidation in tomatoes under water stress, increasing ascorbate and reducing glutathione, differently from non-treated plants (Nasibi et al., 2011). The foliar application of arginine has been proven to increase endogenous phytohormones (auxins, gibberellins and cytokinins) in wheat while reducing abscisic acid (El-Bassiouny et al., 2008). Vílchez et al. (2018) observed the inoculation of pepper plants under drought stress with *Microbacterium* sp. 3J1 resulted in changes to the leaf metabolite profile, specifically affecting the molecules’ concentration in regulating osmotic pressure. Notably, the altered metabolites detected in the inoculated plants exhibited a mirrored response to those detected in *Microbacterium* sp. 3J1 when subjected to drought conditions. Among the metabolites whose abundance increased in inoculated plants, the authors reported arginine; however, it was not upregulated in the microorganism alone when cultivated under water stress. Polyunsaturated fatty acids (PUFA) are essential components of biological membranes, contributing significantly to their structural integrity and fluidity. Moreover, oxygenated PUFA derivatives (oxylipins) serve as bioactive metabolites, that modulate various signal transduction pathways, influencing diverse cellular processes (Savchenko and Dehesh, 2014). Among oxylipins, jasmonic acid (JA) and its immediate precursor, 12-oxophytodienoic acid (OPDA), are the most extensively characterized (Eckardt, 2008). Chromomoric acid is a 12-oxophytodienoic acid metabolite. By the observations documented by Leporino et al. (2024), which reported an increased level of chromomoric acid B in tomatoes treated with protein hydrolysates, thereby enhancing recovery from drought stress, the modulation of fatty acids in plants inoculated with the rhizomicrobiome from *J. phoenicea* may have led to a change in membrane composition, consequently influencing cellular redox status.

Steviol and amaranthine were reduced in plants inoculated with the rhizomicrobiome from *J. phoenicea* compared to all the other treatments. In an analysis of the effect of microbial biostimulants on maize metabolism under drought, Othibeng et al. (2022) found steviol glycosides to accumulate in the plant sap. The authors suggest that the microbial biostimulants trigger the active transport of these molecules to other plant tissues, where they are likely hydrolyzed into sugars and steviol, the latter of which can then be converted into gibberellins. Amaranthine is a pigment found in *Amaranthus* and is known for its bioactive activity. In a study conducted to select *Amaranthus* genotypes for increased amaranthine content, Gins et al. (2002) found in the enriched cultivar Valentina that amaranthine biosynthesis was negatively correlated to leaf lignin, protein, and cellulose content and leaf density. The authors suggest a link between amaranthine biosynthesis and nitrogen metabolism, potentially with amaranthine as an intermediate in cellular nitrogen compound conversion. These results align with our research, in which a decreased abundance of amaranthine was observed in plants inoculated with the rhizomicrobiome from *J. phoenicea*, resulting in an increased number of leaves compared to the other treatments.

Moreover, as shown in Supplementary Figure S3, inoculation with the rhizomicrobiome from *J. phoenicea* significantly increased diterpenoids belonging to the gibberellin class, which are plant hormones that regulate various developmental processes. Both rhizomicrobiomes from *J. phoenicea* and *P. lentiscus* stimulated the production of tryptophan alkaloids, classified as simple indole alkaloids, which include auxin-related compounds. Besides, the abundance of compounds belonging to the classes of sphingolipids and steroids, such as sphingosine-1-phosphate, sphinganine-1-phosphate, and 19-hydroxytestosterone (Supplementary Table S3) decreased following inoculation with rhizomicrobiomes from *J. phoenicea* and *R. officinalis*. Inoculation with rhizomicrobiomes from *J. phoenicea* and *P. lentiscus* resulted in a significant reduction in compounds associated with diterpenoids (3 beta,15,16-trihydroxydolabrene), sesquiterpenoids (artemisinin), and tryptophan alkaloids (pumiloside). A significant reduction in the relative abundance of 19-hydroxytestosterone (−1.25-fold) was also observed in plants inoculated with the rhizomicrobiome from *P. lentiscus*, and the relative abundance of 3 beta,15,16-trihydroxydolabrene decreased by 1.5-fold in the leaf metabolome of plants inoculated with the rhizomicrobiome from *R. officinalis*. Compounds related to the four classes mentioned above are involved in defense mechanisms and signaling processes mediating stress responses (Tholl, 2015; Wang et al., 2018; Mamode Cassim et al., 2020; Liu et al., 2021; Mohammadi-Cheraghabadi and Hazrati, 2023). Their decrease is part of a more complex alteration in the leaf sage metabolism induced by microbiome transplantation. It can be postulated that the reduction of specific metabolites is associated with increased utilization as precursors for other metabolites or decreased synthesis due to re-routing their precursors toward alternative pathways. Interestingly, the unique inoculum responsible for reducing the relative abundance of all six shared metabolites was the rhizomicrobiome from *J. phoenicea*. This specific rhizomicrobiome was the only one contributing to increased root biomass (Figure 4). The inoculation with this rhizomicrobiome influenced several metabolic pathways, primarily affecting one compound from each class. The total number of upregulated metabolites was significantly lower than that observed in the leaf metabolome of plants inoculated with the rhizomicrobiomes from *P. lentiscus* and *R. officinalis*. Moreover, the results reported in Supplementary Table S2 demonstrated that the inoculation did not result in excessive upregulation, with increases ranging from 2- to 3-fold compared to non-inoculated plants. Inoculation with the rhizomicrobiome from *P. lentiscus* led to the accumulation of four phenylpropanoids and two flavonoids, with the flavone diosmetin accumulating up to 5.7-fold more than in non-inoculated plants (Supplementary Table S2). In contrast, inoculation with the rhizomicrobiome from RO resulted in the accumulation of seven distinct flavonoids, three of which exhibited increases between 3.7 and 5.1-fold. A significant correlation between the genetic distance of rhizosphere microbial communities and the phylogenetic distance of host plant genotypes was observed (Bouffaud et al., 2014). This indicates that the evolutionary history of a plant genotype influences the selection of bacterial taxa and shapes the rhizosphere microbiota (Lei et al., 2019). We can speculate that *R. officinalis*-derived rhizometabolome might have triggered a less pronounced response in the closely related *S. officinalis* compared to *J. phoenicea* and *P. lentiscus* microbiomes, highlighting that donor and recipient plant’s phylogeny can influence the response to microbiome transplantation.

In evaluating the effectiveness of microbial inoculants in enhancing stress tolerance, certain studies have employed shoot biomass as the sole indicator (Schmitz et al., 2022). However, Monohon et al. (2021) demonstrated that inoculated plants can exhibit reduced biomass despite developing drought-resistant traits. This phenomenon was attributed to a microbially induced drought avoidance strategy, highlighting the potential for morphological changes prioritizing water conservation more than growth. While acknowledging the value of biomass estimation as a metric for evaluating growth promotion in fast-growing species, it is essential to recognize its limitations in accurately reflecting the benefits of microbial inoculation in slow-growing or stress-tolerant species. Moreover, reliance on biomass measurements can be misleading, particularly in short-term experiments. Based on the observations made by Garcia et al. (2018) on potato plants infected with *Phytophthora*, metabolomics could facilitate the early detection of stress symptoms in asymptomatic plants. The results obtained from this study demonstrate that a comprehensive understanding of plant responses to transplanted microbiomes requires an integrated approach, which includes biomass assessment, physiological analysis, and metabolomics. In cases where biomass remains unchanged, the metabolic adjustments induced by microbial consortia in plants can only be effectively analyzed through a multifaceted approach.

## 5 Conclusion

This study demonstrates that inoculation with microorganisms can induce significant changes in plant morphology, physiology, and resource allocation, significantly influencing plant responses to drought. Notably, inoculation with the rhizomicrobiome from *J. phoenicea* led to increased root biomass, potentially enhancing water and nutrient uptake. This treatment also induced a reduction in leaf size, which may improve thermoregulation, reducing oxidative stress. Furthermore, inoculation with rhizomicrobiomes from *J. phoenicea* and *P. lentiscus* resulted in distinct stomatal conductance patterns, suggesting altered water-use strategies. Metabolomic analysis revealed that microbiome transplantation induced substantial reprogramming of the leaf metabolome. Inoculation with all three rhizomicrobiomes led to the accumulation of secondary metabolites associated with stress tolerance, including flavonoids, phenylpropanoids, and apocarotenoids. Specifically, the rhizomicrobiome from *J. phoenicea* triggered a more balanced metabolic response, with moderate upregulation of a diverse range of metabolites. These results highlight the importance of using a multidisciplinary approach to evaluate the efficacy of selected transplanted microbial communities in enhancing plant stress tolerance, especially in slow-growing species in which differences in biomass production in the short term might not occur. This approach holds promise for the selection and application of microbiomes in revegetation programs and sustainable agriculture in semiarid Mediterranean ecosystems facing increasing water scarcity due to climate change. Further research is needed to fully elucidate the relation between plant responses and the microbiome composition, understand the mechanisms involved and explore the potential applications of these findings in sustainable agriculture and ecosystem management.

## Data Availability

The original contributions presented in the study are included in the article/Supplementary material, further inquiries can be directed to the corresponding authors.
