# Supplementary material for: Soil microbiome transplantation to enhance the drought response of Salvia officinalis L
Source: Front Microbiol. 2025 Mar 12;16:1553922. doi: 10.3389/fmicb.2025.1553922 (PMC11937098; doi:10.3389/fmicb.2025.1553922)
Supplement: Supplementary file 2 [file Data_Sheet_1.pdf]

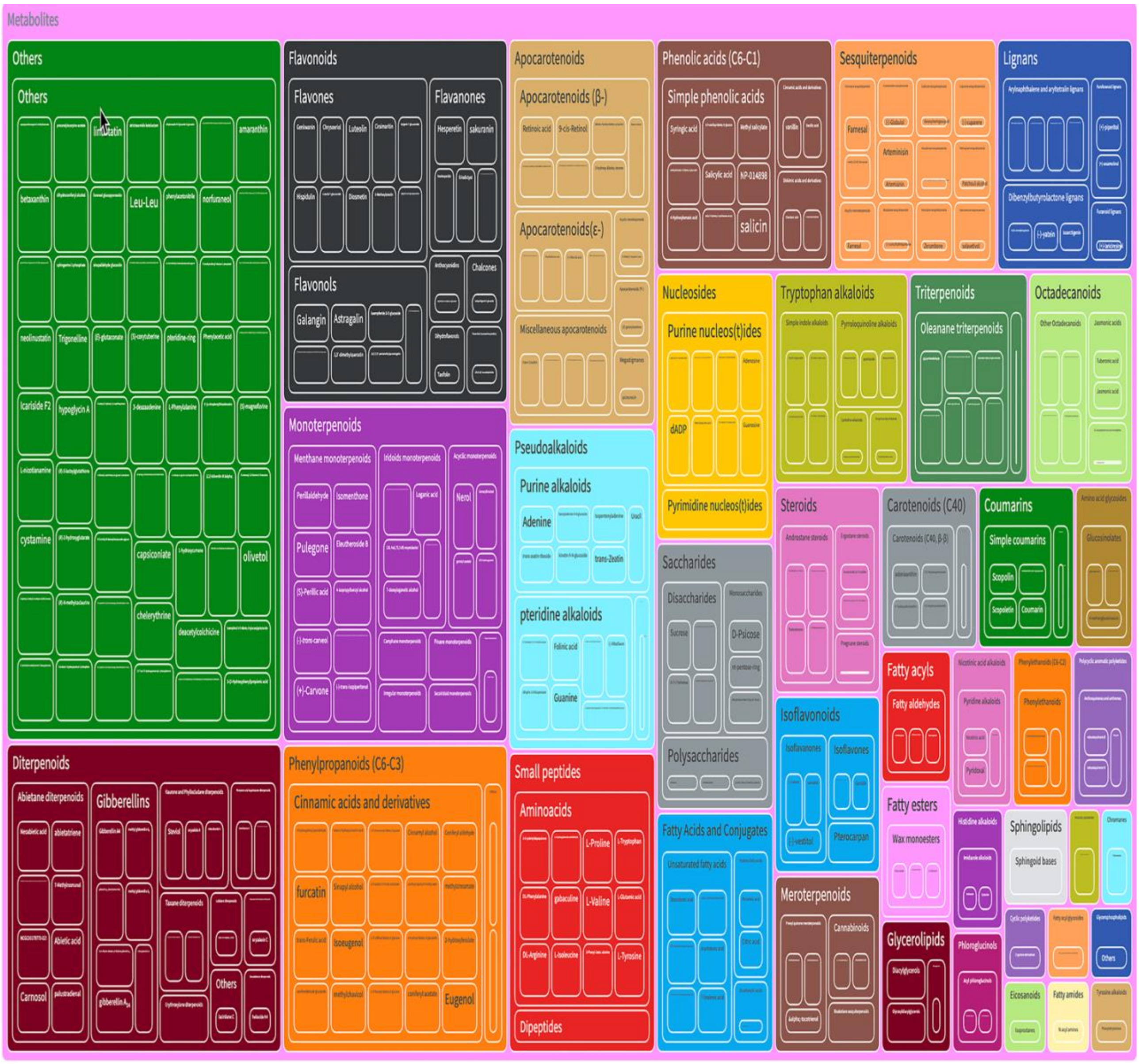

Supplementary Figure S1. Superclasses and classes of metabolites detected in the leaf metabolome of non-inoculated and inoculated plants.

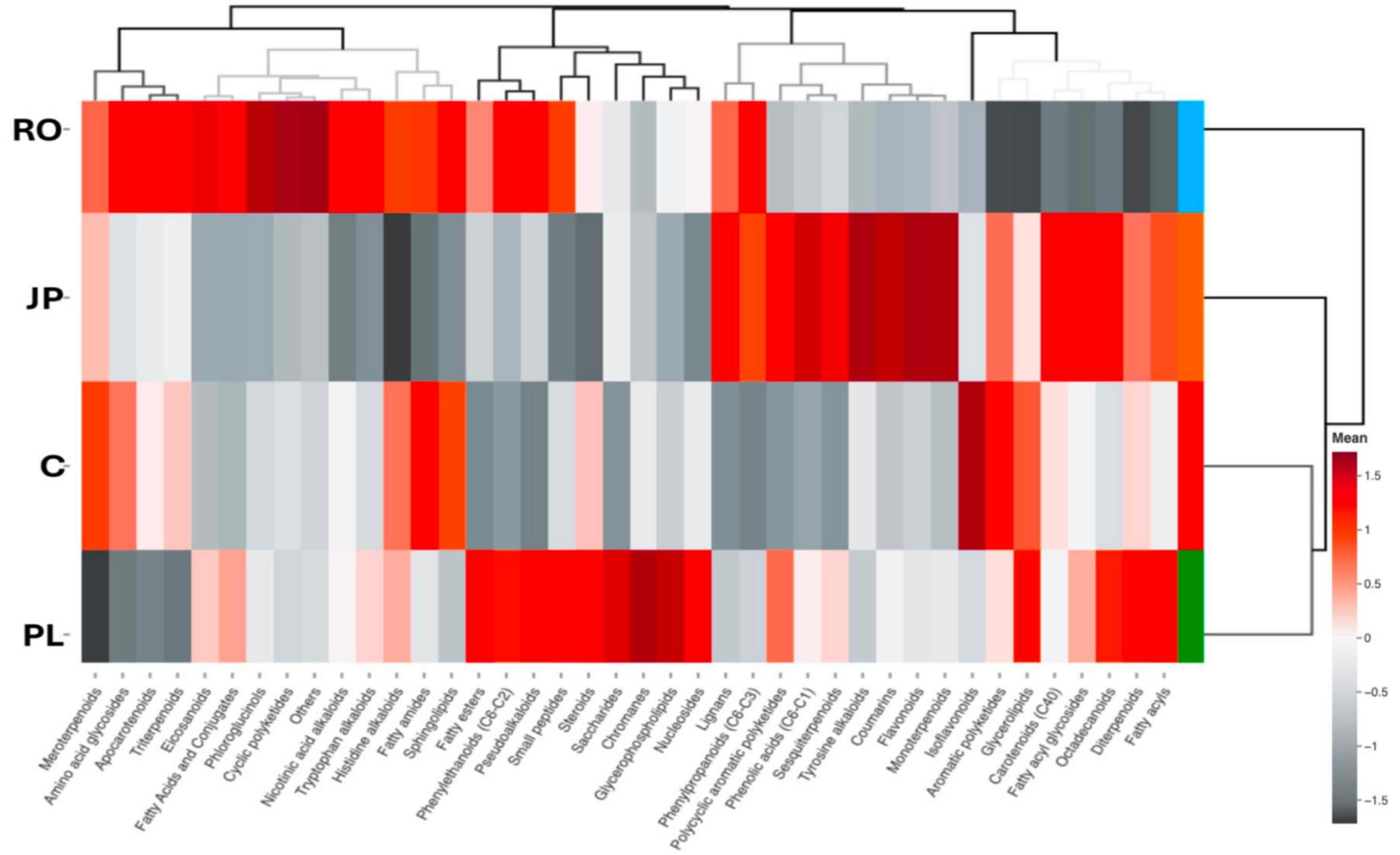

Supplementary Figure S2. **Hierarchical representation of the clusters in the leaf metabolome datasets of non-inoculated plants (C) and plants inoculated with the rhizomicrobiomes from JP, PL, and RO**. (C), non-inoculated sages. (JP), (PL) and (RO), plants inoculated with the rhizosphere microbiomes from *J. phoenicea* L., *P. lentiscus* L., and *R. officinalis* L., respectively.

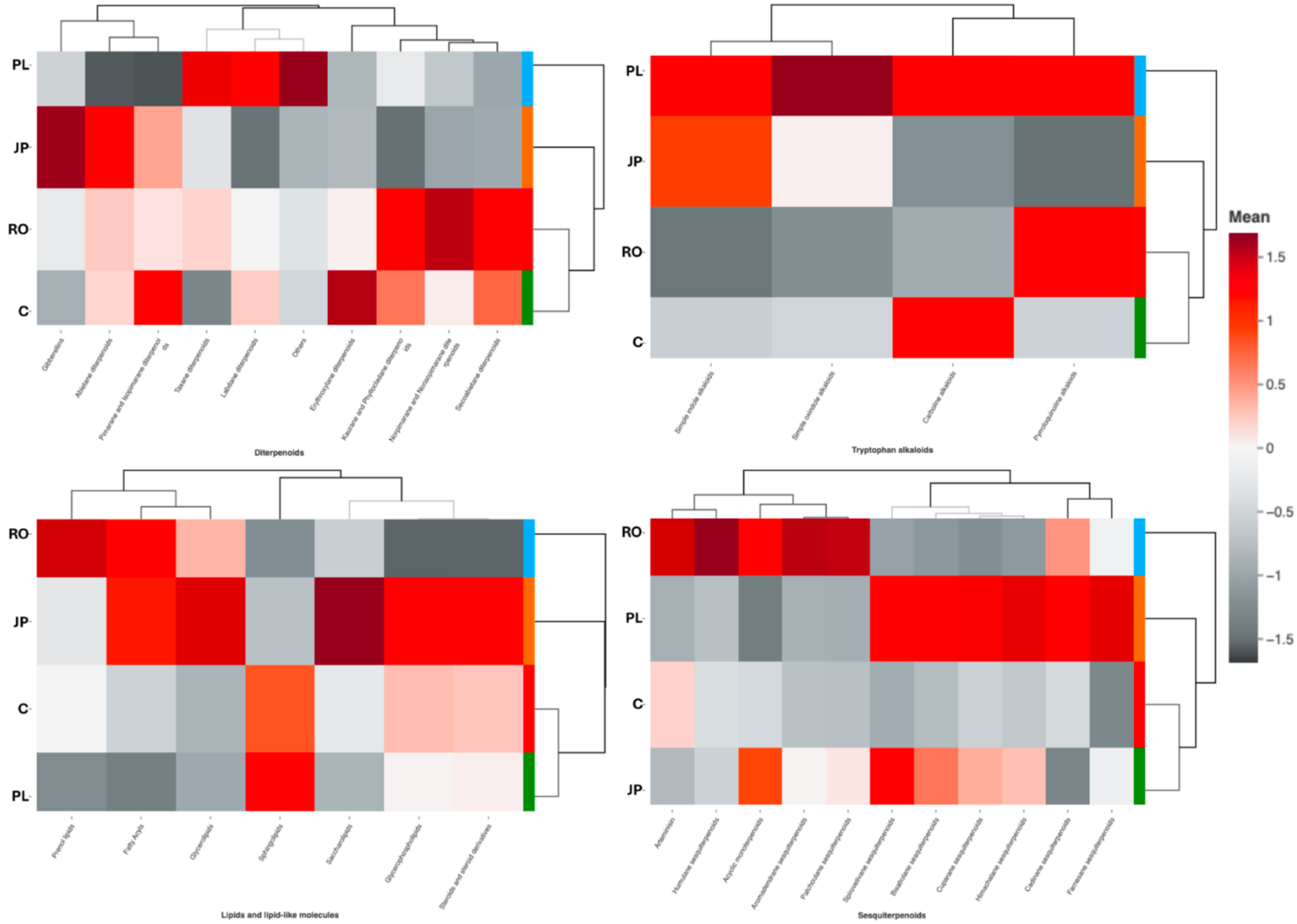

Supplementary Figure S3. **Hierarchical representation of the clusters of diterpenoids, tryptophan alkaloids, lipids and related compounds, and sesquiterpenoids in plants inoculated and non-inoculated by rhizomicrobiome transplanting.**
